# A case report of an emergency in the scrotum of a 4-month-old boy: A hair in the spermatic cord

**DOI:** 10.1097/MD.0000000000008907

**Published:** 2017-12-08

**Authors:** Cong Shang, Jin Zhang, Qian Wang, Li Zhang

**Affiliations:** a Pediatric Surgery; b Pediatrics, Qingdao Women and Children's Hospital, Liaoyang Road; c Department of Ultrasound; d Department of Pathology, The Affiliated Hospital of Qingdao University, Jiangsu Road, Qingdao, China.

**Keywords:** foreign body, pediatric, spermatic cord, ultrasonography

## Abstract

**Rationale:**

Retained foreign body (FB) in soft tissues of children is commonly encountered in the emergency room. The FBs include different kinds of material and associate with some nonspecific symptoms. They usually penetrate the skin or other soft tissues and show some entry holes.

**Patient:**

A 4-month-old male infant was admitted to our department due to the edema on the right of scrotum, higher skin temperature, and a funicular lump with serious haphalgesia in the right groin and scrotum.

**Diagnoses:**

Imaging studies revealed a foreign body in right spermatic cord. A surgical exploration was performed, and a hair was dislodged. The pathological diagnosis was hyperplastic fibrous connective tissue and inflammatory reaction.

**Interventions:**

A surgical exploration was performed to dislodge the foreign body.

**Outcomes:**

The boy had an uneventful course after the surgery and was discharged from the hospital one week later. The incision healed well when the boy came back for follow-up one week later.

**Lessons:**

We still have no idea that how this FB got to the soft tissue. Few previous studies can be retrieved to help us. But we can learn that foreign body should be one of consideration for the origin of inflammation or infection of soft tissues even there is not any laceration and penetration injuries.

## 1 Introduction

Retained foreign body (FB) in the soft tissue is commonly encountered in the emergency room and is an common result of household accidents and child abuse.^[1,2]^ FB is a complication of traumatic soft tissue injuries, including laceration and penetration injuries.^[3]^ FBs may be various, including wood, glass, metal, plastic, or gravel.^[3,4]^ Because many FBs are asymptomatic and radiolucent, their detection is occasionally difficult.^[3]^ Diagnosis of foreign body penetration can be made by obtaining an accurate history, performing a thorough physical examination, and utilizing radiological imaging methods. On admission, the reports from patients and relatives can vary according to the foreign body insertion site.^[5,6]^ Moreover, a physician should realize that sometimes young children cannot readily express their complaints.^[7]^ About 38% of FBs are initially overlooked and may result in inflammation and infection.^[8]^ The purpose of this case report is to present a case of a child with a foreign body that was not identified until we performed an operation. This foreign body has not been reported in previous literatures. His clinical manifestations were inflammation, infection, and pain but without laceration and penetration injuries. To date, we still do not know how the FB penetrated the soft tissue.

## 2 Case presentation

The parents of the patient consented to use the clinical and radiological data of the patient for the publication. This study was approved by medical ethical committee of The Affiliated Hospital of Qingdao University, Qingdao University, Qingdao, China.

A 4-month-old man infant was brought to our emergency pediatrics by his parents. His mother and grandparents who regularly cared for the child during the day denied any trauma history. The history obtained from his mother revealed that the symptoms were paroxysmal dysphoria, swelling, heat, and haphalgesia of the right scrotum for about 2 days. No fever was experienced. The child had no history of medical or surgical treatments, and he was healthy otherwise. Positive physical signs were observed with the edema on the right of scrotum, higher skin temperature, and a funicular lump (4cm × 1 cm) with serious haphalgesia in the right groin and scrotum.

**Figure 1:**
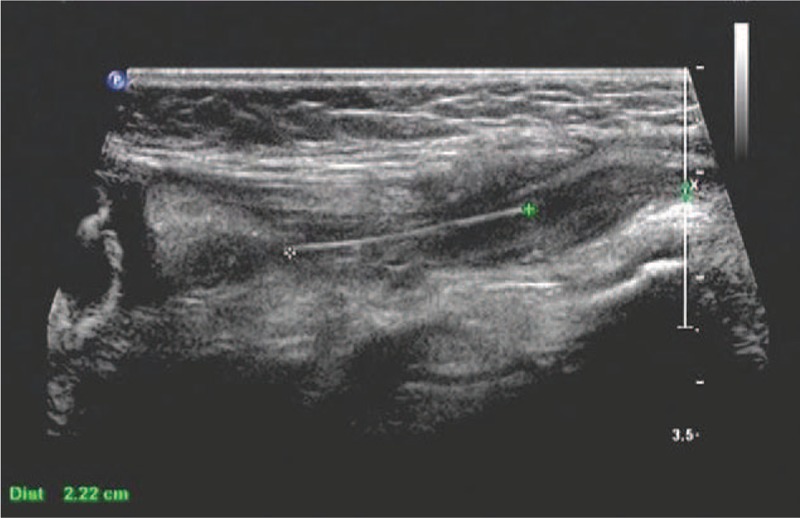
B-type US revealed a threadlike dense echo about 2.2 cm in the right groin. The ultrasound doctor considered that it was a sewing needle. US = ultrasonography.

**Figure 2:**
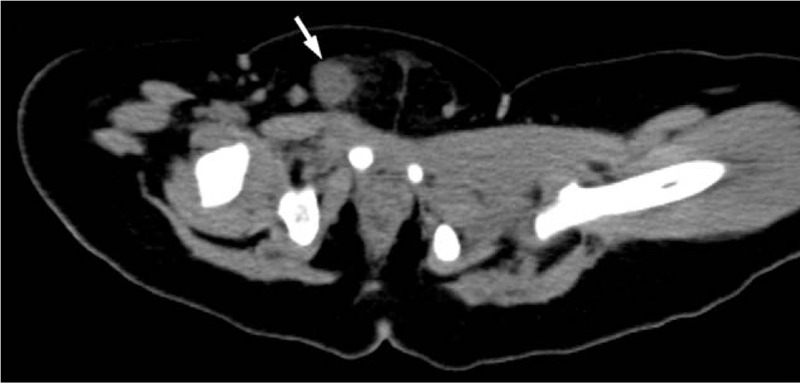
CT examination was performed. No radiopaque FB were found in CT views but the hypertrophic spermatic cord (arrow). CT = computer tomography, FB = foreign body.

**Figure 3:**
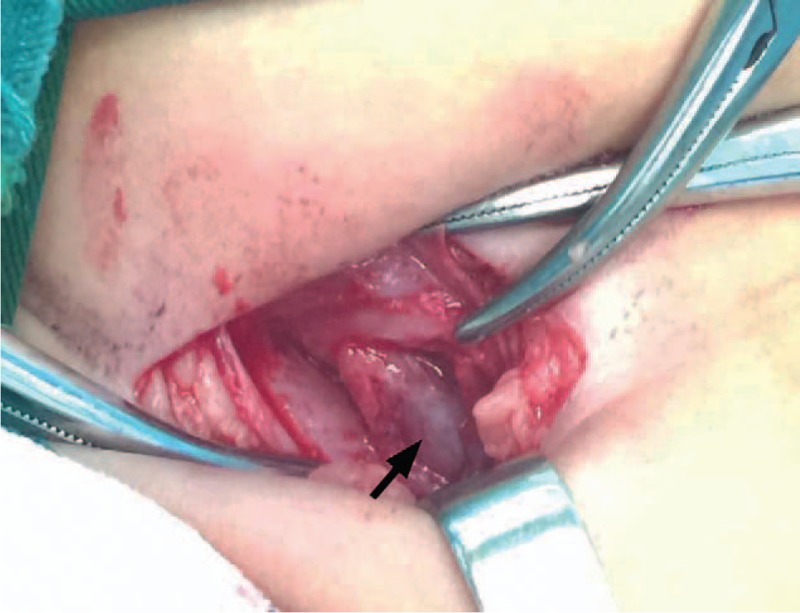
A surgical exploration of the soft tissues was performed. The right spermatic cord was found to be edematous and brittle (arrow).

**Figure 4:**
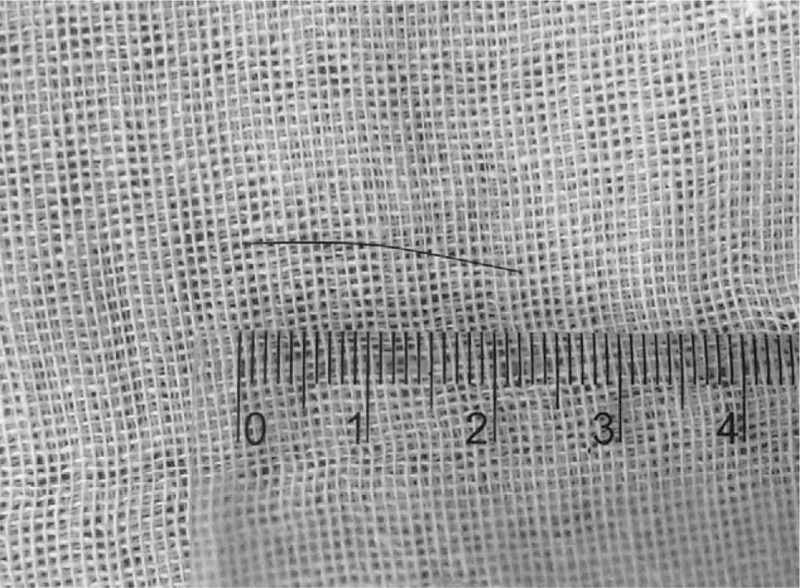
A hair measuring about 2.2 cm was now dislodged when we slivered the right funicular lump.

**Figure 5:**
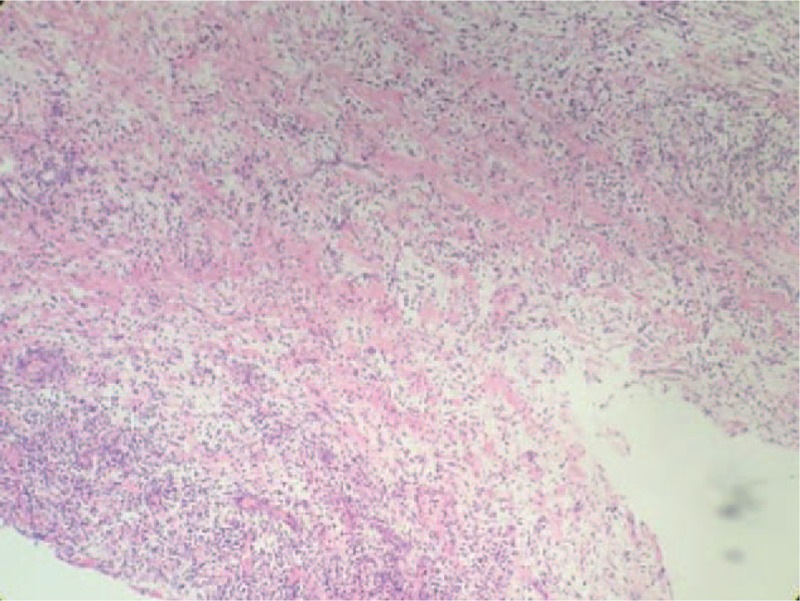
The pathological diagnosis of the part of edematous soft tissues was hyperplastic fibrous connective tissue and inflammatory reaction.

After hospitalization, the findings of his general laboratory examination were within normal limits. Then, imaging examinations were conducted. The B-type ultrasonography (US) revealed a threadlike dense echo about 2.2 cm in the right groin (FB? sewing needle?). The right spermatic cord was hypertrophic as a result of inflammation. The sizes and blood streams of both testes were normal (Fig. 1). In order to explore the particular position of the FB, a computer tomography (CT) examination was performed. The CT views did not show a radiopaque FB but did show the hypertrophic spermatic cord (Fig. 2). Infant abuse was not considered because the boy was always cared by his mother and grandparents who did not have a psychiatric history. A surgical exploration of the soft tissues was performed. During the operation, the right spermatic cord was found to be edematous and brittle (Fig. 3). A hair without hair follicle measuring about 2.2 cm was dislodged when we slivered the right funicular lump (Fig. 4). A part of the soft tissue that surrounded the hair was taken for pathological examination. The boy had an uneventful course after the surgery and was discharged from the hospital 1 week later. The incision healed well when the boy came back for follow-up 1 week later. The pathological diagnosis was hyperplastic fibrous connective tissue and inflammatory reaction (Fig. 5).

## 3 Discussion

Pediatricians frequently encounter children with FBs retained inside soft tissues. FB injuries may be asymptomatic or the symptoms may be nonspecific. The symptoms which usually present themselves as a secondary inflammatory reaction occurs 2 to 3 weeks later after the FB injury and depend on the anatomical location of the injury. The most noticeable symptoms are allergies and infections such as partial edema, haphalgesia, and mild skin lesions. However, the absence of specific symptoms indicating the occurrence of a FB injury can lead to delays in diagnosis. Difficulty of diagnosis will also increase when the patient has forgotten the traumatic incident. This is true more so for a pediatric case, thereby increasing the risk of complications.^[9,10]^ In the present case, the symptoms of the boy were edema and haphalgesia in the scrotum without any skin lesion such as needle entry hole. According to his medical history and positive physical signs, we initially diagnosed him with testicular torsion. But this diagnosis could not explain the funicular lump with serious haphalgesia in the right groin.

The diagnosis of FBs depended on traumatic incident, skin lesion, and imaging examination. X-ray imaging usually should be performed for diagnosing and exploring an anatomical location. Metallic foreign bodies can be seen on x-ray images, but plastic, wooden, and some other radiolucent foreign bodies cannot be seen on x-ray images nor CT images. So US is effective in the diagnosis of radiolucent foreign bodies.^[11]^ In the present case, the FB was detected by US and the inflammatory reaction of circumambient soft tissue was detected by CT images.

FBs include a wide variety of objects. Hard FBs such as wood, glass, metal, plastic, or gravel are so hard that they can penetrate the skin or other soft tissues easily and show some entry holes. However, soft objects usually become FBs in aerodigestive tract by ingesting or inhaling.^[12]^ In the present case, no skin lesion was found, and it was almost impossible that this FB (a hair) could penetrate soft tissues. We have a few conjectures about how this hair got into spermatic cord: the hair from a ruptured teratoma crossed the patent processus vaginalis and then stayed here. Hairs can usually be found in mature teratomas which are germ cell tumors originating from testes and ovaries containing at least 2 of the 3 germ layers. Some extragonadal teratomas, such as anterior mediastinum, retroperitoneum, and sacrococcygeal region, are also reported in the literature.^[13]^ Hairs usually mixed with calcific and nervous tissues in mature teratomas. But in this case, the hair appeared in the right spermatic cord without any other abnormal tissues such as nervous, curtaneous, and skeletal structures. The pathological report suggested the final diagnosis was hyperplastic fibrous connective tissue and inflammatory reaction. Moreover, no intraabdominal or testicular teratoma was found by CT examination before the operation. Some cells from ectoderm stayed here during the development. If so, there may be more tissues including skin and other appendages (sweat gland, sebaceous gland, or nail) also from ectoderm in this location. Meanwhile, this hair should have a complete formation. But in this case, only a single hair without follicle and tip was found finally. This is an ingrown hair with destroyed follicle or an ingrown hair cyst. The hair grows inside the skin and burrows in the uppermost dermis, which is called “ingrown hair.” Patients usually had some clinical manifestations such as inflammation, infection, and pain, just like the boy has in this case. These symptoms may be a possibly reaction stimulated by the hair inside the skin. But in the present case, the hair without follicle and tip was much deeper in soft tissue than the ingrown hair. Ingrown hair usually was found in thick hair area, but this 4-month-old man infant had no hair in his perineum.

Though the FB in this case was very soft and small, it brought pain to the child. Since few previous studies have reported similar FBs, we could not get useful information from those studies. Our conjectures cannot reasonably explain this case, so it still puzzled us about how it got into the soft tissue. US is the best choice for the detection of FBs including radiolucent and radiopaque materials in soft tissue and has a sensitivity and specificity of more than 90%.^[14,15]^ Thus, US-guided FB removal from soft tissue can be chosen firstly because it is inexpensive, repeatable, and less risky. When the FB is too small or deep in the soft tissue or US-guided FB removal fails, surgical removal can be performed subsequently.

## References

[1] Hambrick E Rao TR Lim LT Jejunoaortic fistula from ingested seamstress needle. Arch Surg 1979;114:732–3. 454156 10.1001/archsurg.1979.01370300086016

[2] Sbokos CG Azariades M Chlapoutakis E The removal of sewing needles from two children's hearts. Thorac Cardiovasc Surg 1984;32:373–5. 6084333 10.1055/s-2007-1023426

[3] Budhram GR Schmunk JC Bedside ultrasound AIDS identification and removal ofcutaneous foreign bodies: a case series. J Emerg Med 2014;47:e43–8. 24685452 10.1016/j.jemermed.2014.01.033

[4] Callegari L Leonardi A Bini A Ultrasound-guided removal of foreign bodies: personal experience. Eur Radiol 2009;19:1273–9. 19153745 10.1007/s00330-008-1266-5

[5] Yeung Y Wong JK Yip DK A broken sewing needle in the knee of a 4-year-old child: is it really inside the knee? Arthroscopy 2003;19:E18–20. 10.1016/s0749-8063(03)00745-x 14551567

[6] Nadkarni UM Munshi A Damle SG Retrieval of a foreign object from the palatal root canal of a permanent maxillary first molar: a case report. Quintessence Int 2002;33:609–12. 12238693

[7] Phillips D Walling AD An unusual cause of hip pain in a child. Postgrad Med 1988;84:56–8. 3186566 10.1080/00325481.1988.11700491

[8] Anderson MA Newmeyer WL 3rd Kilgore ES Jr Diagnosis and treatment of retained foreign bodies in the hand. Am J Surg 1982;144:63–7. 7091533 10.1016/0002-9610(82)90603-1

[9] El Bouchti I Ait Essi F Abkari I Foreign body granuloma: a diagnosis not to forget. Case Rep Orthop 2012;2012:439836. 23259122 10.1155/2012/439836 PMC3505897

[10] Vega Curiel A Villaverde Romon M Carrillo Lucia F Injuries from palm tree thorn simulating tumoral or pseudotumoral bone lesions. Acta Orthop Belg 2001;67:279–82. 11486692

[11] Blankstein A Cohen I Heiman Z Ultrasonography as a diagnostic modality and therapeutic adjuvant in the management of soft tissue foreign bodies in the lower extremities. Isr Med Assoc J 2001;3:411–3. 11433632

[12] Jotdar A Dutta M Kundu S Nasopharynx- the secret vault for lost foreign bodies of the upper aerodigestive tract. Iran J Otorhinolaryngol 2006;28:431–3. PMC5168576 28008395

[13] Hui JPK Luk WH Siu CW Teratoma in the region of an adrenal gland in a 77-year-old man. JHK Coll Radio 2004;l7:206–9.

[14] Jacobson JA Powell A Craig JG Wooden foreign bodies in soft tissue: detection at US. Radiology 1998;206:45–8. 9423650 10.1148/radiology.206.1.9423650

[15] Bray PW Mahoney JL Campbell JP Sensitivity and specificity of ultrasound in the diagnosis of foreign bodies in the hand. J Hand Surg Am 1995;20:661–6. 7594298 10.1016/S0363-5023(05)80287-4

